# Validity and reliability of the Approaches to Dementia Questionnaire (ADQ) in Indonesian health students

**DOI:** 10.1186/s12909-025-07332-2

**Published:** 2025-05-29

**Authors:** Sri Mulyani, Gary Mitchell, Gillian Carter, Bob Woods, Sri Warsini, Azam David Saifullah, Aisyah Iffah Ulayya, Christine Brown Wilson

**Affiliations:** 1 Department of Mental Health and Community Nursing, Faculty of Medicine, Public Health, and Nursing, Universitas Gadjah Mada, Yogyakarta, Indonesia; 2 School of Nursing and Midwifery, Queen’s University Belfast, Belfast, UK; 3 School of Health Sciences, Bangor University, Bangor, UK; 4 School of Nursing, Faculty of Medicine, Public Health, and Nursing, Universitas Gadjah Mada, Yogyakarta, Indonesia

**Keywords:** Approaches to Dementia Questionnaire (ADQ), Attitude, Dementia, Health Professions Student, Reliability, Validity

## Abstract

**Background:**

Individuals living with dementia often visit healthcare settings, so it is important for health professionals to have appropriate dementia care training. A key component of dementia care is a positive, person-centred attitude towards people with dementia. As future healthcare workers, health students need to develop positive attitudes early in their education. To assess and support the development of such attitudes, a brief, valid, and reliable tool is needed in Indonesia. However, no such tool is currently available in Indonesia. The Approaches to Dementia Questionnaire (ADQ) is a well-established instrument that measures care staff attitudes and has been shown to predict staff behaviour and the recognition of people with dementia. Therefore, this study aims to translate, adapt, and assess the validity and reliability of the Indonesian translation of the modified ADQ for use with health students.

**Methods:**

This methodological study was conducted from October to November 2023 to adapt the modified ADQ into Indonesian. The translation process followed established cross-cultural adaptation by Brislin guidelines, including translation, synthesis, expert review, and testing. Two translators from different language institutions translated the instrument into Indonesian (T1 & T2). These two versions were synthesised into an integrated version (T12) by a panel of experts. The final instrument was tested on 161 fourth-year nursing, medical, and health nutrition students recruited through consecutive sampling.

**Results:**

The Indonesian version of the ADQ demonstrated an overall internal consistency of 0.584 (Cronbach's alpha), which is considered acceptable. Subscale reliability was moderate for the Hope subscale (α = 0.552) and higher for the Personhood subscale (α = 0.701). Item-total correlation values ranged from 0.261 to 0.588, indicating moderate validity overall; however, three items (Items 1, 6, and 15) were identified as invalid with correlation coefficients below 0.195.

**Conclusion:**

The Indonesian version of the ADQ demonstrated lower reliability and variable subscale consistency compared to adaptations in other countries. This may be due to limited public awareness and associated stigma towards dementia. These findings highlight the need for further refinement of item wording and better alignment with local contexts, to improve the tool’s validity and reliability for use in different populations.

## Background

Dementia is the primary cause of dependency and disability in older persons [1]. Globally, over 55 million people have been diagnosed with dementia [2]. This number is projected to reach 78 million in 2030 and 139 million in 2050, doubling every 20 years [2]. The rise of dementia prevalence will mostly occur in Low- Middle-Income countries (LMICs) [2], where dementia research is frequently scarce or nonexistent [3]. According to Alzheimer's Disease International (2019), LMICs account for about 60% of the 10 million individuals who are diagnosed with dementia annually [1], and this is possible to rise to 71% in 2050 [2]. However, it should be noted that the coverage of dementia diagnosis in LMICs only ranges from 5 to 10% [4]. In comparison, high-income countries (HICs) may diagnose around 50% of dementia cases with established dementia services [5]. LMICs face several unique challenges that contribute to the underdiagnosis of dementia, such as limited financial resources for healthcare [3, 6], restricted assessment methods [3, 7], insufficient access to care [7] and conflicting agendas within the healthcare system [6]. There is also wide variation of economic and socio-cultural differences, significant differences in service accessibility between rural and urban areas, as well as additional risk factors for different ethnic groups and Indigenous communities [8]. Although Indonesia has been an Upper- Upper-middle-income country (UMIC) since 2023 [9], this country also faces challenges similar to those faced by LMICs when considering dementia services and care. In Indonesia it is estimated that 1.2 million people in Indonesia had dementia in 2016 [10]. By 2030, that figure is likely to rise to 2 million; by 2050, it is predicted to reach 4 million [10].

Dementia cases are often commonly found in healthcare settings, including primary care [11], general hospitals [12] and acute hospitals [13]. However, in primary care, studies show that only about 25–40% of dementia cases are identified, with some diagnosed only in the late stages [11]. In hospital settings, mild dementia is often underreported, as it may not significantly impact treatment and is not consistently recognised as a chronic condition [14]. Additionally, dementia patients face many negative consequences during hospitalisation, including an increased risk of behavioural and psychological symptoms, delirium, falls, greater dependence on caregivers, cognitive and functional decline, malnutrition, dehydration, poor health outcomes, and higher rates of harm and mortality [15]. Despite the growing concern over dementia, Indonesia lacks fundamental data on its prevalence and the challenges it presents across healthcare settings.

In Indonesia, misconceptions and the lack of understanding of dementia are frequent [16] accompanied by prominent stigma [17], such as the emergence of terms such as"crazy"or"insane"referring to people with dementia [18]. Most Indonesians believe dementia is a normal part of ageing and are unfamiliar with the terms dementia or Alzheimer's [16]. A study with over 200 participants examining the general public's attitudes in Yogyakarta, Indonesia, towards patients with Alzheimer's was largely pessimistic [19]. In contrast, a survey of 4220 people in Jakarta and North Sumatra, Indonesia, showed that most participants exhibited mixed attitudes and tended not to be negative [16]. However, a study of 334 nursing students [20] showed that nursing students’ attitudes were lower than those of 386 nurses at the largest teaching hospital [21].

Although there were various findings related to attitudes toward dementia in Indonesia, it is crucial to continue to raise awareness of dementia and promote positive attitudes towards dementia. A greater understanding of dementia may develop positive attitudes, significantly reducing unfriendly behaviours toward people with dementia [22]. Developing a positive attitude towards dementia may help one feel more at ease and confident while engaging with others with the disease [23], which can enhance both the quality of care they get and their quality of life [24]. In contrast, negative attitudes can disrupt the effectiveness of communication, contributing to inadequate services and forming a stigma that hinders understanding and compassion for people with dementia [25]. Stigma may create social isolation for people with dementia and their caregivers and even provoke physical violence against those with dementia [18]. In Indonesia, stigma mainly impacts family members due to the religious and cultural obligations expected of them in undertaking caring responsibilities [26].

Given the above urgency, all health professionals must have awareness, knowledge, and a positive attitude, regardless of their future discipline or speciality. These three things must be obtained during a student's health professional learning period. As a part of the healthcare team during clinical placement, health professions students have responsibilities for caring for people with dementia [27], including managing challenging behaviours, such as agitation, aggression, refusal to care, and disorientation [28]. In addition, students in health professions are crucial in disseminating information about dementia to the general public [29]. Studies on health professions student attitudes towards people living with dementia have been conducted in several countries, including Indonesia [20], Uganda [29], Brazil [30], China [31], Malaysia [23], and Malta [32]. All studies show that students in health professions, primarily nursing and medicine, demonstrate positive attitudes toward people with dementia. However, compared to health professionals, health students tended to have lower attitude scores [33, 34]. In Indonesia, focused dementia education in health professional programs is lacking [35], potentially resulting in the development of negative attitudes toward dementia. Therefore, it is essential to evaluate health students'attitudes, not only to assess the impact of training programs and other interventions. To accommodate this need, a brief, valid, and reliable measurement tool is needed to determine attitudes towards dementia.

Various questionnaires measure attitudes towards dementia. One questionnaire that has the potential for cross-cultural adaptation and is in line with the objectives of this study (to evaluate the effects of a digital dementia awareness game) is the Approaches to Dementia Questionnaire (ADQ). The ADQ has been widely used in previous research [25, 36–41] and has proven valid and reliable for general and special populations. Research with care staff indicated that the ADQ comprised two factors, labelled'hope'and'recognition of personhood.'A more'hopeful'attitude predicted more positive care staff behaviour, whereas a more'person-centred'attitude was associated with greater recognition of dementia in patients [42]. The ADQ has been previously modified to measure the general public's attitudes toward people with dementia using an online survey method [37].

This study is part of a broader research programme that initially developed a digital game in the United Kingdom (UK) to promote public understanding of dementia by emphasising the remaining abilities of individuals with dementia [43–45]. Building on the success of the UK project, a similar game was codesigned and culturally adapted for use in Indonesia, where public knowledge of dementia is still limited. To assess the effectiveness of this intervention in the Indonesian setting, the current study aims to translate, adapt, and validate a modified version of the ADQ. This tool will be used to evaluate public attitudes towards dementia before and after playing the digital game. In addition, it will encourage cross-cultural comparisons of how digital interventions influence dementia-related attitudes across different countries, including the UK [36, 45] and Indonesia. The constructs of the ADQ also reflected the game’s development in terms of hope and personhood in dementia care. Thus, psychometric testing of the ADQ in the Indonesian context is a critical step in ensuring the cultural relevance and accuracy of the educational tool.

As a fundamental step in this research programme, it is pivotal to conduct psychometric testing of the ADQ on health professional students, a population for which this has not yet been carried out in Indonesia, is necessary. This study aims to assess the validity and reliability of the ADQ in health professions students at a primary health sciences faculty in Yogyakarta, Indonesia. The validated and reliable of the ADQ's Indonesian version will subsequently be used to evaluate changes in attitudes before and after playing with a culturally adapted digital dementia awareness game as an intervention developed as a continuation of the UK co-design study.

## Methods

### Design

This methodological study tests the ADQ's construct validity and reliability (internal consistency). It was conducted from October to November 2023 at the Faculty of Medicine, Public Health and Nursing, Universitas Gadjah Mada.

### Participants

This study involved a total of 161 valid respondents. The required minimum sample size was calculated by multiplying the number of ADQ items (19 items) by a minimum ratio of 1:6 [46], resulting in 114 participants being recruited. An additional 10% was added to account for potential dropouts or unusable responses, leading to the target minimum sample size of 126.

To achieve the target sample size, this study employed a consecutive sampling method with inclusion criteria of active nursing, medical, and nutritionist students from the 2020 intake (4 th year) willing to participate voluntarily and had access to a smartphone, computer, or tablet with internet. The exclusion criteria are students on annual leave (absent from lectures over a year) when data collection occurred and were not completing a health-related degree.

Researchers recruited respondents by approaching the class leaders of each year's group in each study program via WhatsApp. Afterwards, researchers sent posters and Google form links to the class leaders to distribute to the class group. The online questionnaire remained open for two months (October to November 2023). A total of 174 responses were initially received. Of these, 13 responses were identified as duplicates (students who filled out the form more than once) and were excluded. No incomplete responses were found, as the online form required all items to be completed before submission. Thus, the final sample included 161 complete and valid responses.

### Instrument

The ADQ, or Approaches to Dementia Questionnaire, gauges attitudes regarding dementia. This instrument shows the degree to which a person-centred approach is supported and the individual's expectations regarding dementia. In England and Wales, 123 nursing home employees participated in developing the ADQ, including seven home managers, 29 registered nurses, 26 care assistants with National Vocational Qualifications (NVQ), and 61 care assistants without formal training [42]. A 2016 modification of the ADQ employing an online survey approach to measure public attitudes toward individuals with dementia revealed a Cronbach's alpha of 0.86 [37].

The 19 Likert scale items of the ADQ have five possible responses:"Strongly Disagree,""Neutral,""Agree,""Strongly Agree,"and"Disagree."Additionally, it has two subscales: Hope and Recognition of Personhood (RoP). Eight items [1–4, 6, 8, 10, 13] comprise the Hope subscale, which measures optimism or pessimism regarding the future and capacities of individuals with dementia. Eleven items (5, 7, 9, 11, 12, 14, 15, 16, 17, 18, and 19) make up the RoP subscale, which measures how much a person understands people with dementia as distinct individuals who share the same values or how much they comprehend person-centeredness. Higher total ADQ scores indicate a more positive attitude toward persons with dementia, ranging from 19 to 95. The Hope subscale ranges from 8 to 40, while the RoP subscale has scores ranging from 11 to 55.

The ADQ has been widely used in previous research, is valid and reliable, and can be used for the general population [37, 45], dementia care staff [40, 41], and health professions students [43, 44]. Compared to other attitude questionnaires, such as Dementia Care Attitude Scale (DCAS) [31], Adolescent Attitudes towards Dementia Scale (A‐ADS) [47], Dementia Attitude Scale (DAS) [48], Bryan’s Dementia Attitude Scale (BDAS) [49], and Attitudes Toward Aggression Scale (ATAS) [50], the ADQ has items that use simple and easy-to-understand language. In addition, the ADQ is suitable for measuring attitudes in medical students [51].

In Indonesia, questionnaires measuring attitudes regarding dementia have been used in several previous studies, such as DAS on nursing students [20] and BDAS on the general population [19]. DAS reflects three components of attitudes (affection, behaviour, and cognition) toward individuals with Alzheimer’s disease and related dementias [52]. In contrast, BDAS consists of two factors, namely optimism and pessimism [19]. However, the ADQ considers hope and personhood both aspects integral to supporting people living with dementia. The ADQ has never been used to measure attitudes toward dementia in Indonesia.

It should be noted that other researchers had previously adapted and conducted psychometric tests using the Indonesian version of the ADQ when this research was being conducted. However, the results of the psychometric tests have yet to be published. During the research process, the researcher communicated with the author to determine whether the translations were comparable.

First, the cross-cultural adaptation process started with translating the modified ADQ by Cheston et al. [37] by two translators from different language institutions who did not know each other and had no medical or clinical background (T1 & T2). The panel of experts, consisting of gerontological, community, and mental health nursing experts from Universitas Gadjah Mada, conducted face-to-face discussions to compare T1 and T2 and evaluated the differences between the two versions of the questionnaire. This evaluation was started by identifying discrepancies, such as whether one version misses the meaning of the original instrument or whether one uses terminology appropriate in the healthcare context. The experts used consensus among the team to agree on the best version of each item, but sometimes also combined the two translations or reworded entirely for clarity and cultural fit to the Indonesian context. For instance, the experts chose to use the phrase “*Orang dengan demensia” instead of “Pengidap demensia” or “Orang-orang yang mengalami demensia”* because this phrase is common in Indonesia and briefer. Experts also considered retaining conceptual equivalence over literal translation, ensuring the meaning or idea behind the item remains the same even if the exact word changes. Last but not least, one of the team members documented the justification for specific wordings. As a result, an integrated version (T12) of the translated ADQ was formed in the second stage. Finally, the researchers then compared the synthesised version (T12) with an existing Indonesian version of the ADQ translated by another researcher (Woro, unpublished). The comparison revealed that both versions were broadly similar, with no fundamental difference in meaning or interpretation. Therefore, in this paper, we only report the translation and adaptation process conducted in the current study. Based on this comparison, the prefinal version of the Indonesian ADQ was finalised. In this study, as an existing translated version with similar content was available (Woro), the back translation step was omitted. Moreover, pre-final testing was not performed due to time and resource constraints.

### Data collection

Data collection was carried out online using Google Forms. The class leader shared the questionnaire link via their WhatsApp groups, and follow-up reminders were sent every 3 to 7 days. In the questionnaire link, prospective respondents consented to complete the questionnaire before continuing to fill out the demographic details and then the ADQ, which took approximately 5 to 10 min to complete.

### Data analysis

Data were cleaned before analysis to ensure no duplicate responders using participant cell phone numbers. Of the 174 respondents who filled out the questionnaire, 13 respondents filled out the questionnaire more than once, so the final data analysed was 161. The researchers utilised Google Forms features only to obtain completed responses from participants, evading missing data.

Researchers tested construct validity using Pearson product-moment [53–55] and assessed the findings using its critical value coefficient (r > 0.195) [54] as well as factor analysis [56]. Pearson's correlation coefficient quantifies the linear relationship between quantitative variables. It is frequently used to confirm the strength of the current linear association between variables [53]. At the same time, factor analysis aims to investigate the underlying dimensions that explain interactions between complex variables or items [57]. In this study, researchers tested internal consistency reliability using Cronbach's alpha. The numerical variable description table used the mean and SD for normally distributed data and the median and minimum–maximum data for non-normally distributed data. Researchers used SPSS version 26 IBM Corp. (Armonk, NY, USA) for data analysis.

## Results

### Respondent characteristics

Data were collected from 161 Faculty of Medicine, Public Health, and Nursing Universitas Gadjah Mada students. In this study, the majority of respondents were women (83.3%) and were completing medical study programs (67%) (Table 1). The median age of respondents was 21 years, with the youngest 19 years and the oldest 23 years. The majority of respondents did not have family with dementia (81.4%), had never interacted with people with dementia (67.1%), and had never cared for people with dementia (90.7%). Only five of 161 respondents had attended dementia training.

**Table 1:** Characteristics of respondents in ADQ validity and reliability testing, Indonesia, October–November 2023 (*n* = 161)

Characteristics	n (%)	Median (Minimum–Maximum)
**Age (years)**		21 (19–23)
**Gender**		
Women	134 (83.2)	
Men	27 (16.8)	
**Study Program**		
Nursing	59 (36.6)	
Health Nutrition	37 (23.0)	
Medicine	65 (40.4)	
**Have a family with dementia**		
Yes	30 (18.6)	
No	131 (81.4)	
**Interaction with people with dementia**		
Yes	53 (32.9)	
No	108 (67.1)	
**Caring for people with dementia**		
Yes	15 (9.3)	
No	146 (90.7)	
**Previous dementia training**		
Yes	5 (3.1)	
No	156 (96.9)	

In this study, a subset analysis was conducted to determine whether there are differences in the validity and reliability of the ADQ based on demographic characteristics, as recommended in previous studies [58]. Table 2 shows that the group of respondents aged 21 years and females had a higher Cronbach alpha value because they had a larger sample size. In contrast, the group of respondents who were health nutrition students with experience caring for people with dementia and who had dementia training had a higher Cronbach alpha even though the sample size was smaller. In addition, the group of respondents who had a family history of dementia and had interacted with people with dementia tended to show almost the same Cronbach alpha value.

**Table 2:** Validity and reliability for each characteristics of respondents in ADQ, Indonesia, October–November 2023 (*n* = 161)

Characteristics	r-value	Overall Cronbach’s Alpha
**Age (years)**		
19 years old (*n* = 1)	n/a	n/a
20 years old (*n* = 14)	−0.328–0.923	0.443
21 years old (*n* = 117)	0.097–0.602	0.642
22 years old (*n* = 28)	−0.229–0.621	0.301
23 years old (*n* = 1))	n/a	n/a
**Gender**		
Women (*n* = 134)	0.062–0.621	0.607
Men (*n* = 27)	−0.212–0.612	0.464
**Study Program**		
Nursing (*n* = 59)	0.029–0.589	0.548
Nutrition (*n* = 37)	0.073–0.672	0.614
Medicine (*n* = 65)	−0.130–0.612	0.598
**Have a family with dementia**		
Yes (*n* = 30)	0.088–0.569	0.559
No (*n* = 131)	0.036–0.601	0.588
**Interaction with people with dementia**		
Yes (*n* = 53)	0.024–0.530	0.564
No (*n* = 108)	0.035–0.596	0.598
**Caring for people with dementia**		
Yes (*n* = 15)	−0.065–0.692	0.686
No (*n* = 146)	0.059–0.621	0.573
**Previous dementia training**		
Yes (*n* = 5)	−0.159–0.841	0.695
No (*n* = 156)	0.052–0.581	0.589

### Validity and reliability test results

In this study, Table 3 displays each item's Pearson correlation coefficient values, revealing that items 1, 6, and 15 were invalid because their respective r-values were less than the table of r-values for the correlation coefficient. Table 3 also shows a moderate Cronbach's alpha of 0.552 for the hope subscale and a relatively high one of 0.701 for the personhood subscale [59]. Meanwhile, the overall Cronbach alpha was 0.584, which is considered acceptable or moderate [59, 60].

**Table 3:** Validity and reliability test results of the ADQ version in Indonesian, Indonesia, October–November 2023 (*n* = 161)

Item	Brief Descriptors of Each Item and the ADQ version in Indonesian	R-value	Interpretation of Validity	Cronbach's Alpha
**Hope Subscale**				**0.552**
1	Strict routine	**0.043**	**Invalid**	
	*Penting untuk memiliki rutinitas yang sangat ketat saat menangani orang dengan demensia*			
2	Like children	0.281	Valid	
	*Orang dengan demensia sangat mirip dengan anak-anak*			
3	No hope	0.588	Valid	
	*Tidak ada harapan untuk orang dengan demensia*			
4	Can’t make decisions	0.451	Valid	
	*Orang dengan demensia tidak mampu membuat keputusan sendiri*			
6	Sick	**0.113**	**Invalid**	
	*Orang dengan demensia itu sakit dan perlu dirawat*			
8	Nothing can be done	0.396	Valid	
	*Tidak ada yang bisa dilakukan untuk orang dengan demensia, kecuali menjaga mereka tetap bersih dan nyaman*			
10	Downhill inevitable	0.288	Valid	
	*Begitu demensia berkembang pada diri seseorang, tidak bisa dihindari bahwa kondisi mereka akan semakin memburuk*			
13	Too attached	0.396	Valid	
	*Penting untuk tidak terlalu terikat pada seseorang dengan demensia*			
**Recognition of Personhood (RoP) Subscale**				**0.701**
5	Activities	0.466	Valid	
	*Penting bagi orang dengan demensia untuk tetap aktif dan terlibat dalam hal-hal yang mereka suka*			
7	Choices	0.287	Valid	
	*Penting bagi orang dengan demensia untuk diberikan sebanyak mungkin pilihan dalam kehidupan sehari-hari mereka*			
9	Understanding and reassurance	0.261	Valid	
	*Orang dengan demensia lebih cenderung merasa puas ketika diperlakukan dengan penuh pengertian dan diberikan ketentraman*			
11	Need for respect	0.444	Valid	
	*Orang dengan demensia perlu merasa dihormati, sama seperti orang lain*			
12	Psychological needs	0.422	Valid	
	*Untuk mencapai kualitas hidup yang baik bagi orang dengan demensia, kita harus mempertimbangkan kebutuhan psikologis, sosial, dan fisik*			
14	Doesn’t matter what you say	0.516	Valid	
	*Tidak masalah dengan apa yang Anda katakan kepada orang dengan demensia karena mereka akan melupakannya*			
15	Good reasons for behaviour	**0.175**	**Invalid**	
	*Orang dengan demensia sering memiliki alasan yang masuk akal untuk melakukan tindakan yang mereka lakukan*			
16	Enjoyable to be with	0.355	Valid	
	*Menghabiskan waktu bersama orang dengan demensia sangat menyenangkan*			
17	Empathy and understanding	0.482	Valid	
	*Penting untuk menanggapi orang yang mengalami demensia dengan empati dan pengertian*			
18	Can do a lot	0.489	Valid	
	*Ada banyak hal yang dapat dilakukan oleh orang dengan demensia*			
19	Special understanding	0.290	Valid	
	*Orang dengan demensia hanyalah manusia biasa yang membutuhkan perhatian khusus untuk memenuhi kebutuhan mereka*			
**Total ADQ**				**0.584**

The r-value refers to the Pearson correlation between each item and the total score

Next, the researchers examined every ADQ item's response distribution to determine whether the ADQ Indonesian version has floor or ceiling effects (F/C). Table 4 illustrates that there were three items where more than 40% of respondents responded"Strongly Agree", indicating these items had notable ceiling effects [61]. Conversely, there were no items where more than 40% of participants chose to"Strongly Disagree,"indicating no floor effects in this study [61].

**Table 4:** Item completeness and response distribution for the ADQ Indonesian version, Indonesia, October–November 2023 (*n* = 161)

Item	N	% Missing	Response Category (%)				
			**Strongly Agree**	**Agree**	**Neither Agree or Disagree**	**Disagree**	**Strongly Disagree**
ADQ1	161	0	23.6	51.6	17.4	7.5	0
ADQ2	161	0	10.6	57.8	18.6	13.0	0
ADQ3	161	0	0.6	3.1	15.5	58.4	22.4
ADQ4	161	0	2.5	44.7	27.3	23.6	1.9
ADQ5	161	0	**49.7**	47.2	3.1	0	0
ADQ6	161	0	15.5	52.2	24.8	7.5	0
ADQ7	161	0	9.9	49.7	27.3	13.0	0
ADQ8	161	0	2.5	14.9	16.8	60.2	5.6
ADQ9	161	0	24.2	67.7	8.1	0	0
ADQ10	161	0	3.7	40.4	39.8	15.5	0.6
ADQ11	161	0	36.6	59.6	3.7	0	0
ADQ12	161	0	**48.4**	50.3	1.2	0	0
ADQ13	161	0	1.2	11.8	35.4	44.7	6.8
ADQ14	161	0	0	5.6	9.9	58.4	26.1
ADQ15	161	0	0.6	28.0	53.4	16.1	1.9
ADQ16	161	0	2.5	14.9	73.3	8.7	0.6
ADQ17	161	0	**46.0**	50.9	3.1	0	0
ADQ18	161	0	13.7	65.8	14.3	6.2	0
ADQ19	161	0	25.5	63.4	8.1	3.1	0

*Abbreviation*: *ADQ* Attitude toward Dementia Questionnaire

Thereafter, the factor analysis test results in Table 5 illustrate that the factor loading of the Indonesian version of ADQ items was the same as the factor loading in the original version of ADQ. In addition, the factor model generated in the Indonesian version of ADQ is explained by 30.53% (moderate) [57].

**Table 5:** Factor analysis of the ADQ Indonesian version, Indonesia, October–November 2023 (*n* = 161)

Item	Factor Loading	
	**Hope**	**RoP**
ADQ1	0.072	
ADQ2	0.588	
ADQ3	0.588	
ADQ4	0.633	
ADQ5		0.554
ADQ6	0.291	
ADQ7		0.318
ADQ8	0.527	
ADQ9		0.544
ADQ10	0.472	
ADQ11		0.719
ADQ12		0.699
ADQ13	0.414	
ADQ14		0.516
ADQ15	0.065	
ADQ16		0.405
ADQ17		0.762
ADQ18		0.434
ADQ19		0.501

*Abbreviation*: *ADQ* Attitude toward Dementia Questionnaire, *RoP* Recognition of Personhood

## Discussion

This is the first psychometric evaluation of the modified ADQ in Indonesia involving three experts. It has been tested on 161 nursing, health nutrition, and medical students. The results of this study obtained an r value ranging from 0.261 to 0.588, with three items (1, 6, and 15) identified as invalid. The overall Cronbach's alpha value was 0.584, indicating low reliability, with a moderate value of 0.552 for the hope subscale and a higher value of 0.701 for the personality subscale.

Regarding the results, a reliability level ranging from 0.5 to 0.707 is still acceptable if other high construct indicators are included in the exploratory study [62]. These requirements can be applied in this study since the personhood subscale has a high alpha value (0.701). Hence, the overall alpha scale meets acceptable thresholds for exploratory research. Nevertheless, the instrument should be revised and refined to meet higher reliability for confirmatory research.

Reflecting on the findings in this study, it can be concluded that the Cronbach alpha is lower than those of previous psychometric results using the ADQ [37, 63–68]. The variance in results could be due to differences in respondents in terms of sample size and characteristics (having interactions with people affected with dementia, having a family with dementia, previous dementia training), research contexts (including culture), the cultural adaptation process of the instrument (questionnaire translation). Besides, the lower reliability of the Indonesia version of the ADQ in this current study might be affected by the lack of awareness of dementia in Indonesia. Prior research on ADQ adaptation has primarily been conducted in countries where dementia awareness campaigns are well established. By contrast, in Indonesia, people still frequently have misconceptions and lack information regarding dementia [16], even though a public awareness campaign is a key priority of Indonesia’s National Strategy 2015 [64].

The ADQ has demonstrated higher reliability in various settings than other studies. The ADQ has been adapted into the Chinese version by Leung et al. in 2013 [39]. An experienced medical translator translated it, and a psychiatrist back-translated the ADQ. Both were fluent in Chinese and English [39]. Subsequently, an expert panel consisting of three psychogeriatricians, one specialist psychogeriatric nurse, and one occupational therapist specializing in geriatrics assessed these instruments and agreed on their relevance in the Hong Kong healthcare system [39]. However, the Chinese version of ADQ has not been tested for its internal consistency in Hong Kong, with potential for emerging cultural deviation in understanding the items [39]. Although the Chinese version of the ADQ was tested in a pilot study [39], it was unclear how many respondents were included or what the respondents’ criteria were [39]. The Chinese version of ADQ was then used to examine the attitudes of staff across different sites and populations: these included 1,780 staff in residential care homes for the elderly (RCHE) in Hong Kong [39]; evaluation of a dementia training program on 140 home care staff’s attitudes in Taiwan [65], and to compare the ADQ with the Sense of Competence in Dementia Care Staff-Chinese version (SCIDS-C) on 187 nursing homes, 82 care assistants, and 205 health professionals from 12 nursing homes and three departments in a general hospital in China [66]. The Chinese version of ADQ had a Cronbach's alpha of 0.72 in Taiwan [65] and 0.74 in China [66]. However, there are limitations with these studies, for example, in the Taiwan study, it is unclear whether the researchers used different samples for psychometric testing or the primary samples in their controlled study [65]. If they used the same samples as the main research, this study does not identify which data they used to test the reliability, whether baseline (T0), week 12 (T1), and week 24 (T2), or using intervention or control group [65]. Alternatively, if they did not use respondents similar to those in the primary study, it is unclear how many respondents they used and their criteria. Meanwhile, in the China study, the Chinese version of the ADQ has been reported to demonstrate positive but weak correlations with the SCIDS-C (r = 0.22, p-value < 0.001) in the concurrent validity test [66]. The higher reliability of the ADQ in the Chinese version compared to the Indonesian version may be a result of the higher number of respondents in the Taiwan and China studies. Moreover, healthcare professional participants would have more experience caring for dementia patients, potentially resulting in better knowledge and attitudes toward dementia than health students in this current study.

In Japan, a study examined the effect of the Behaviour Analytics & Support Enhancement (BASE) program on 46 care managers and professional caregivers of home care services, using the Japanese version of the ADQ to evaluate their attitudes [67]. The Japanese version of the ADQ was adapted by Suzuki et al. [68] and demonstrated good internal consistency with Cronbach's alpha of 0.83 [67]. However, it is challenging to to explore the psychometric testing process of the Japanese version of the ADQ as the paper is not written in English [68].

The English version of the ADQ demonstrates high internal consistency in multiple studies in Ireland [69] and the UK [37, 63, 70]. A study in Ireland showed that the ADQ was reliable, with a Cronbach's alpha of 0.78 for the overall score, 0.70 for the subscale hope, and 0.77 for the person-centered subscale [69]. This study involved 906 doctors, nurses, and healthcare attendants; 210 allied professionals; and 667 general support staff. Reflecting on the sample's criteria in the Ireland study, it is evident that most of them were healthcare professionals. Moreover, as many as 23.5% of the sample had previous dementia training, and 37.9% of the sample had a family member with dementia [69], these characteristics could increase the reliability of the ADQ in the Ireland study.

Furthermore, research in the United Kingdom (UK) on 77 healthcare staff caring for older people from nine inpatient wards across two large National Health Service (NHS) Trusts demonstrated that the ADQ had good psychometric properties with a Cronbach's alpha of 0.83 and test–retest reliability of 0.76 [70]. Most participants have ≤ 5 years (34.2%) and 6–15 years (34.2%) time working with dementia. These demographicsmay contribute to the higher Cronbach’s alpha and test–retest reliability compared to this current study. In this current study, most respondents (81.4%) had no family members with dementia, 67.1% had never dealt with someone who had dementia, and 90.7% had never provided care for someone who had dementia., which may account for the variance in results.

Further studies in the UK with 794 respondents in the Bristol and South Gloucestershire area showed good internal reliability of the ADQ with a Cronbach’s alpha of 0.86 (95% Confidence Interval (CI) 0.85–0.87) and alphas of 0.77 (95% CI 0.76–0.79) for the Hope and 0.84 (95% CI 0.83–0.85) RoP subscales, respectively [37]. In this study, the researcher modified six items (three in the Hope subscale and three in the RoP subscale) [37]. The researcher changed the phrase “dementia sufferers” in items 1 and 6 with “people with dementia” and slightly changed the wording of items 5, 12, 13, and 19 [37]. This study excluded respondents who have worked with people affected with dementia to represent the lay public’s attitudes and involved 49.7% of those with dementia [37]. The ADQ-modified version in this study was then used on a further study on 2,201 people in the Bristol and South Gloucestershire area by online form (*n* = 1865) and written form (*n* = 337) to investigate whether attitudes toward dementia are correlated with having experience of dementia (either as a result of working with dementia or by being impacted by dementia) [63].

Finally, research on 316 dementia care staff recruited from a dementia training organisation, four Cognitive Stimulation Therapy (CST) training events, and seven residential dementia care homes, both private and public, in Greater London, UK demonstrated Cronbach’s alpha of 0.78 overall, 0.73 for hope, and 0.74 for the RoP, respectively [71]. As this study included dementia care staff, this possibly contributed to the higher internal consistency than the current research. Furthermore, in this study, the ADQ was compared to the SCIDS, resulting in a positive correlation with the RoP subscale (r(174) = 0.13, p-value = 0.04) but no correlation with the hope subscale [71].

In this study, the hope subscale shows only a moderate Cronbach's alpha of 0.552, whereas the personhood subscale has a relatively high one of 0.701. The"hope"dimension seeks to convey a feeling of optimism or pessimism regarding the future and capacities of those with dementia. On the other hand, the “personhood” dimension of the ADQ shows how people with dementia are recognised and treated as unique persons. The lower alpha value in the hope subscale than the personhood subscale may be caused by stigma and lack of awareness regarding dementia in Indonesia [18]. In this country, there is a belief that people with dementia are crazy [18], impulsive, and dangerous [1], and even a pessimistic opinion toward the diagnosis of Alzheimer's Disease[19]. Instead of encouraging the abilities of people with dementia, stigma may cause people to be more focused on the symptoms of dementia [72]. Therefore, the moderate alpha value in the subscale may stem from stigma and low awareness related to dementia in this study.

Several factors might cause the low reliability of the ADQ version in Indonesian in this study. The first factor is the sample size. Although some experts state that samples should include 1 to 6 respondents per item in research validating measurement instruments [46], the statistical power of the analysis may be insufficient because the sample size is less than 200. Adequate statistical power for data analysis may be achieved if N > 200 [73]. Guilford (1954) supports this statement, stating that 200 samples are the minimum number for Pearson correlation analysis [74].

In addition, factor analysis might be the second factor contributing to low reliability in this study. Floor and ceiling (F/C) effects measure the sensitivity and coverage of a questionnaire at each end of the scale. They are defined as the percentage of respondents earning the greatest (ceiling) or lowest (floor) possible score across any given domain [75]. Inadequate questionnaire performance can be indicated by high F/C effects, which can also imply restricted instrument range, measurement inaccuracy, and answer bias [75]. Moreover, F/C effects may lead to the inability to distinguish between respondents at either extreme of the scale [76]. In this current study, it is noticeable that there were three items where more than 40% of respondents responded"Strongly Agree,"highlighting notable ceiling effects [61]. These ceiling effects may cause the Indonesian version of the ADQ to be low in reliability since they can weaken reliability and validity [77]. Ceiling effects can cause the interpretability of the questionnaire to be less valid, such as the results of research found in the Netherlands regarding psychometric testing that tested tools for evaluating the quality of care from the patient's perspective [78]. These effects may occur in a survey that measures perceptions and opinions because the instrument’s sensitivity is limited, resulting in the inability to gain the survey respondents’ proper perspectives [79]. Conversely, there were no items where more than 40% of participants chose to"Strongly Disagree,"indicating no floor effects in this study [61].

The third factor that might influence the low reliability of the ADQ questionnaire in this study is the low motivation of respondents. Previous research suggests that high-quality survey results can be produced from motivated participants to optimise the response process [80, 81]. One of the factors that influences respondents to a survey is their willingness to respond. Survey participants driven to finish the questionnaire tend to give more thorough and precise responses [81]. Numerous elements, including the degree of sensitivity and difficulty of the questions, attitudes toward finalising the survey, and views regarding social surveys, are linked to respondent motivation. Suppose participants are not inclined to fill out the questionnaire. In that instance, there is no guarantee that the estimates will be accurate and valid, even though the respondent has received the survey request. Unmotivated survey respondents often choose"good enough"responses to save mental strain and complacency [80], or if they are unwilling to read the question correctly, they typically choose the middle response option [81]. Even when respondents are convinced to participate in a survey, measurement and nonresponse errors could still impact their responses. Expenses, benefits, and trust are needed to motivate respondents to complete the questionnaire [80]. This research took more than two months to get the number of respondents according to the previously planned sample size calculation. Researchers conducted regular follow-ups alongside incentivisation, such as prize draws for randomly selected respondents, to boost the response rate. Although healthcare students in one university in Indonesia were chosen for this study, the lack of dementia training in their programs may suggest a lack of awareness about dementia influencing their responses.

The fourth factor that might influence the low reliability of ADQ is the questionnaire's Likert scale format, which can be challenging for different cultures [82]. One hypothesis is that the idea of measuring something on a continuum is foreign to specific cultures and may be reflected in one or more of the following: (a) Responding to questions and the range of choices on a Likert scale may be more difficult for people from specific cultural backgrounds; (b) People who belong to particular cultural groups may be more inclined to respond in ways that deviate from a Likert scale's response options; (c) Individuals from different cultures may respond to Likert items in different ways. Certain cultures, for instance, may discourage people from choosing extreme reactions; (d) In specific cultural contexts, Likert scale scores may not be as trustworthy; (e) The correlations between variables that are theoretically proposed and assessed using Likert scales may exhibit distinct patterns of association among different cultures. Put differently, a Likert-measured variable's construct validity may be limited to particular cultural contexts [82]. In other ways, the culture of society in Indonesia regarding dementia may influence the low reliability of the ADQ. In Indonesia, there is still a stigma associated with dementia: people are either viewed as insane, or it is connected to spiritual issues, or it is seen as a normal part of ageing [35]. The ADQ may be less reliable than studies conducted in other nations due to the stigma attached to dementia and the general lack of public knowledge about dementia [18, 19].

This study showed that items 1 (r = 0.043) and 6 (r = 0.113) on the hope subscale and 15 on the personhood subscale (r = 0.175) were invalid. If items 1 and 6 are eliminated, the Cronbach's alpha subscale will rise from 0.552 to 0.672. Removing item 15 will decrease the Cronbach's alpha subscale to 0.733 from 0.701. Eliminating items 1, 6, and 15 will result in a 0.672 Cronbach's alpha overall instead of 0.584. However, they were retained because they were deemed necessary due to their content.

Similarly, items 6 and 15 were also found invalid in research conducted on 107 respondents over 18 years working in the intellectual disability sector in Ireland [83]. Research in Ireland demonstrated that items 6, 10, and 15 did not correlate with other items on each scale [83]. In the Irish sample, if items 6 and 10 on the hope subscale were deleted, Cronbach's alpha would be increased from 0.675 to 0.705. If item 15 on the personhood subscale were deleted, Cronbach's alpha would increase from 0.735 to 0.753. Removing these invalid items would increase the ADQ total Cronbach's alpha from 0.749 to 0.770. Although items 6, 10, and 15 were not valid in the Irish study, the ADQ still demonstrated good overall reliability. This result differs from the current research, showing only a total ADQ reliability of 0.672. This suggests that different contexts and samples may influence item reliability. For example, in Indonesia, the level of knowledge about dementia is low, which may explain these responses to items 6 and 15. In Indonesia, dementia awareness public campaigns still prioritise making people aware that dementia or senility is not a normal part of ageing and that it is a disease. Hence, respondents may feel ambiguity toward item 6 (‘people with dementia are sick…’. Besides that, limited awareness related to dementia may create confusion in answering item 15 (there may be good reasons for the behaviour of people with dementia) if respondents do not understand the struggle that people with dementia face in expressing their needs, emotions, or discomfort from their behaviours, even those unusual, complex, and challenging. For instance, a change in routine or a noisy environment may result in distress or agitation in people with dementia. Thus, understanding the individual’s perspective and needs and looking beyond the surface of dementia-related behaviours is crucial to possessing dementia knowledge, ultimately developing a positive attitude.

Item 1 (importance of a strict routine) being invalid could be due to confusion over the reversed item, i.e., the unfavourable answer is reversed in order on the Likert scale. Regarding reversed items, confusion is a response style that varies based on the difficulties of the items and the cognitive skills required to respond accurately to the situation [84]. The poor awareness of dementia in Indonesia [19–21, 85–87] may have contributed to respondents'misunderstanding when answering this question. For instance, students might be aware that dementia is characterised by declines in memory, language, problem-solving, and other cognitive areas that affect a person's ability to carry out daily activities and connect with others [88]. Consequently, the students might think about creating routines that would help people with dementia know what to expect and continue with their everyday activities [89]. However, they may not be aware that these routines should be adapted to each individual's abilities, thus confusing them.

The ADQ is a commonly used scale to estimate a person-centred approach toward individuals with dementia [90]. Just fifteen respondents to this study reported having cared for someone with dementia, which may account for three of the invalid questions. In comparison, these three items were significantly related to daily dementia care. Therefore, limited experience caring for people with dementia may contribute to the invalid three items in this study.

Given the above potential reasons for items 1, 6, and 15's invalidities, some actionable steps can be taken to address this issue. Future research may seek to clarify wording by rewriting items to acknowledge the cultural context of the community's understanding of dementia in Indonesia and ensure respondents understand the meaning behind the question. This step may be helpful to reduce ambiguity, bias, irrelevance, and poor alignment in terms of invalid items. Subsequently, pilot testing and more comprehensive cultural adaptation could be conducted to obtain valid items that measure the same constructs.

Regarding factor analysis, this study exhibited moderate factor loadings of 30.53%. Factor loadings should be at least 0.3 (mild) [57] or 0.4 for interpretation purposes, but the range standard is between 0.5 and 0.7; in other words, the construct should explain at least 25–49% of the variance in each indicator [91]. The factor loadings in this current study were much lower than previous research in Ireland, which had a factor loading of 70.59% [83]. This may be attributed to the different respondents’ characteristics in the two studies. In the Ireland study [83], the participants were 109 people over 18 years old who work with older individuals with intellectual disabilities. Moreover, dementia services and support in Ireland are better than in Indonesia, demonstrated by the existence of the Alzheimer Society of Ireland (the largest dementia-specific provider in Ireland), the landmark Model of Care for Dementia in Ireland [92], and the range of dementia research centres in its universities.

In previous research, the ADQ was used and modified for the home care setting to measure the attitudes of home care nurses in the United States (US) [24]. However, ADQ can be used by the general public [37, 45] and medical students [93]. Furthermore, ADQ has been used to evaluate attitudes after training for dementia caregivers in Germany [94], health professionals in the UK [41], and health professionals working in acute care in Scotland [36]. In the UK, the ADQ has also been used to compare public and health professional students'attitudes regarding dementia before and after they played a digital game [43–45].

The ADQ has the advantage of measuring a person's attitude toward dementia, but this scale has weaknesses. A prior study rated it as generally satisfactory, including 304 employees who worked in acute medical and orthopaedic wards across five District Health Boards in New Zealand [90]. However, it was challenging to distinguish differences in attitudes and confounding factors between diverse respondents, such as levels of knowledge and education. Instead of focusing on attitudes toward practical application, the ADQ more effectively examines knowledge about dementia [90].

As the ADQ assesses the extent to which a person-centred approach is taken toward individuals with dementia, there is potential to use this instrument to improve person-centred care in Indonesia. By identifying healthcare providers’ attitudes toward dementia, the ADQ may discover misunderstandings hindering effective dementia care since there is no person-centred care evaluation tool related to dementia care presently in Indonesia. This instrument can also be utilized to evaluate training or education programs. Insight from the adaptation of the ADQ may be helpful to understanding more personalized approaches for individuals with dementia, ultimately shaping positive attitudes and supporting policy development in countries with limited financial resources, such as Indonesia.

By reflecting on this psychometric study, it can also be learned that several topics should be presented in Indonesia as a curriculum or basis for public campaigns so that the public, especially health professions students, can better understand dementia and build positive attitudes. First, it is imperative to know what dementia is. Dementia is a syndrome caused by several diseases that destroy nerve cells and detriment to the brain, resulting in cognitive function deterioration beyond the common process of ageing and activity daily living performance limitation [95]. Second, people may learn about the signs and symptoms of dementia, including the factors behind it, such as Alzheimer’s, vascular diseases, Lewy Body, Parkinson's disease, etc. Recognizing the underlying reasons for their behaviour can help people respond with patience and compassion rather than frustration. Third, people need to understand that each person with dementia is unique, as they may experience each stage differently and progress at different rates [96]. Understanding this can support people with dementia to maintain or even stimulate their cognitive skills.

In summary, the Indonesian adaptation of the ADQ demonstrated lower reliability (Cronbach's alpha of 0.584) compared to adaptations in other countries, possibly due to limited dementia awareness campaigns and prevalent misconceptions about the condition in Indonesia. The reliability of its subscales varied, with a moderate Cronbach's alpha (0.552) for the hope subscale and a higher value (0.701) for the personhood subscale. Additionally, three items (1, 6, and 15) were deemed invalid in the Indonesian context, a pattern observed in some other countries. These findings appear to support the need for further cultural adaptations of the ADQ, including refining item wording and aligning the instrument with local cultural contexts, to enhance the reliability and validity of the ADQ when applied across diverse settings.

### Limitations and recommendations

This study has several limitations. First, the back translation process was omitted owing to the availability of a comparable existing translation. Furthermore, pre-final testing was not undertaken due to time and resource constraints. Second, this study's sample size is small, even though it used at least 6 participants for each item, so future studies could use larger samples to achieve sufficient statistical power analysis. Third, while the sample includes students from key health disciplines, this study may not fully represent the broader population of health professions students across Indonesia, particularly those from private universities, vocational institutions, earlier years of study, or other health professions. For example, this research was only conducted in a single public university in Indonesia due to logistical and resource constraints. It employed a consecutive sampling method to recruit 161 health profession students. Although the sample was limited to fourth-year students across three academic programs: nursing (36.6%), nutrition (23%), and medicine (40.4%), the decision to do this was due to fourth-year students were able to provide perspectives informed by comprehensive academic and clinical training as they near completion of their program. However, future studies may involve a more diverse sample to enhance generalizability.

In addition, this study only used internal consistency to measure reliability so that further research could use external consistency to estimate the reliability of the ADQ. Moreover, the researcher did not retest after modifying the invalid items so that additional research could retest the invalid items on a larger sample. Finally, this study used an online data collection method, which may result in selection bias (individuals who are more interested in the topic may be over-represented, while others who are less engaged may not participate) or technical barriers (such as loss of power or internet) that may interfere with completion of the questionnaire. As participants completed the questionnaire online independently, the researcher could not observe the respondents'responses while completing the questionnaire. Therefore, further research could collect data directly from respondents, for example, in a classroom context as part of a class where the researcher could be available to answer any queries, which may increase the response rate.

## Conclusions

The Indonesian version of the ADQ can measure health students'attitudes towards dementia, although its reliability is low compared to adaptations in other countries. In addition, three items were invalid but were retained due to their essential content. However, patterns related to invalid items were also observed in several countries. The low reliability and invalid items in the Indonesian version of the ADQ may be due to several factors, such as limited awareness of dementia and prominent dementia stigma in Indonesia, the small sample size of the study, the presence of ceiling effects, online data collection methods, and the Likert scale format. These limitations can be addressed in future research to improve the reliability and validity of the ADQ in diverse settings.

## Data Availability

The datasets used and analysed during the current study are available on the reasonable request from the corresponding author.

## Abbreviations

A-ADS: Adolescent Attitudes towards Dementia Scale
ADQ: Approaches to Dementia Questionnaire
ATAS: Attitudes toward Aggression Scale
BDAS: Bryans’ Dementia Attitude Scale
DAS: Dementia Attitude Scale
DCAS: Dementia Care Attitude Scale
F/C: Floor or Ceiling
HICs: High-Income Countries
LMICs: Low- and Middle-Income Countries
NVQ: National Vocational Qualifications
RoP: Recognition of Personhood
UMIC: Upper- and Middle-Income Country
